# Curcumin Alleviates Diabetic Cardiomyopathy in Experimental Diabetic Rats

**DOI:** 10.1371/journal.pone.0052013

**Published:** 2012-12-14

**Authors:** Wei Yu, Jiliang Wu, Fei Cai, Jizhou Xiang, Wenliang Zha, Dan Fan, Shuang Guo, Zhangyin Ming, Chao Liu

**Affiliations:** 1 Department of Pharmacology, Tongji Medical College, Huazhong University of Science and Technology, Wuhan, China; 2 Hubei Province Key Laboratory on Cardiovascular, Cerebrovascular, and Metabolic Disorders, Hubei University of Science and Technology, Xianning, China; University of Otago, New Zealand

## Abstract

**Objectives:**

Diabetic cardiomyopathy (DCM), characterized by myocardial structural and functional changes, is an independent cardiomyopathy that develops in diabetic individuals. The present study was sought to investigate the effect of curcumin on modulating DCM and the mechanisms involved.

**Methods:**

An experimental diabetic rat model was induced by low dose of streptozoticin(STZ) combined with high energy intake on rats. Curcumin was orally administrated at a dose of 100 or 200 mg·kg^−1^·d^−1^, respectively. Cardiac function was evaluated by serial echocardiography. Myocardial ultrastructure, fibrosis area and apoptosis were assessed by histopathologic analyses. Metabolic profiles, myocardial enzymes and oxidative stress were examined by biochemical tests. Inflammatory factors were detected by ELISA, and interrelated proteins were measured by western blot.

**Results:**

Rats with DCM showed declined systolic myocardial performance associated with myocardial hypertrophy and fibrosis, which were accompanied with metabolism abnormalities, aberrant myocardial enzymes, increased AGEs (advanced glycation end products) accumulation and RAGE (receptor for AGEs) expression, elevated markers of oxidative stress (MDA, SOD, the ratio of NADP^+^/NADPH, Rac1 activity, NADPH oxidase subunits expression of gp91^phox^ and p47^phox^ ), raised inflammatory factor (TNF-α and IL-1β), enhanced apoptotic cell death (ratio of bax/bcl-2, caspase-3 activity and TUNEL), diminished Akt and GSK-3β phosphorylation. Remarkably, curcumin attenuated myocardial dysfunction, cardiac fibrosis, AGEs accumulation, oxidative stress, inflammation and apoptosis in the heart of diabetic rats. The inhibited phosphorylation of Akt and GSK-3β was also restored by curcumin treatment.

**Conclusions:**

Taken together, these results suggest that curcumin may have great therapeutic potential in the treatment of DCM, and perhaps other cardiovascular disorders, by attenuating fibrosis, oxidative stress, inflammation and cell death. Furthermore, Akt/GSK-3β signaling pathway may be involved in mediating these effects.

## Introduction

Diabetes mellitus (DM) has been recognized as a major health problem. The number of diabetic patients worldwide is increasing very fast and expected to reach 439 million by 2030 [1]. The leading cause of death in diabetic patients is cardiovascular disease, which are responsible for the three quarters of the deaths among this population [2]. Diabetic cardiomyopathy (DCM), as an independent diabetic cardiac complication, is characterized by the myocardial dysfunction in the absence of coronary artery disease, hypertension, or valvular heart disease. It has symptoms of early-onset diastolic and late-onset systolic dysfunction [3], which is associated with both type 1 and type 2 DM. Previous studies demonstrated that hyperglycemia, lipid accumulation, excessive generation of reactive oxygen species, cardiac inflammation, accumulation of cardiac fibrosis, and apoptosis are all probably involved in the pathophysiology of DCM [2], [4]. However, the development of DCM has been poorly understood and the mechanisms underlying have not been completely elucidated.

Curcumin, a yellow curry pigment isolated from turmeric powder, is known to be provided with many effects such as antioxidant, scavenging free radicals, anti-inflammatory, anti-tumor, anti-microbial, and so on. Turmeric even has been used to treat DM in ancient Chinese and India. Research in the last two decades has revealed that curcumin can reverse insulin resistance, hyperglycemia, hyperlipidemia, and other symptoms linked to obesity and obesity-related metabolic diseases [5]. Curcumin also has protective effects against diabetic nephropathy [6]. However, the possibility whether curcumin may have beneficial effects in the inhibition of DCM or not has little to be addressed till now.

In the present study, experimental diabetic animal model was induced by low dose of streptozoticin (STZ) combined with high energy intake on rats [7]. We aimed to investigate the potential effects of curcumin on alterations of cardiac function and morphology in diabetic rats, and the associated mechanisms as well.

## Materials and Methods

### Experimental Animals

Seventy-five male Wistar rats (70–90 g) were purchased from Laboratory Animal Center (Hunan, China). Before experiments, all rats were fed with basal diet (BD) for one week. All animals were treated in accordance with the *Guide for the Care and Use of Laboratory Animals* published by the US National Institutes of Health (NIH Publication No. 85–23, revised 1996). All experiments were approved by IACUC (Institutional Animal Care and Use Committee of Huazhong University of Science and Technology) under permit number 2011-S238.

### Induction of Experimental Diabetes on Rats

Experimental diabetic rats were induced by feeding with high fat diet (HFD) during the whole experimental period, whereas rats consuming BD at the same time served as a control group. HFD was prepared by adding 20% sucrose (w/w) and 20% lard (w/w) into BD. After 8 weeks, rats fed with HFD were intraperitoneally injected with STZ (Sigma Aldrich, St. Louis MO, USA) at the dose of 40 mg/kg dissolved in 100 mM citrate buffer pH 4.5 for just 1 time. Control rats were administered an equivalent volume of citrate buffer [8]. Blood glucose levels were measured 72 h after STZ injection using hand-held glucometer (Changsha Sinocare Inc. China) by tail vein puncture blood sampling. Serum triglyceride (TG) and total cholesterol (TC) were determined by auto-biochemical analysis system (OLYPUS AU2700). And body weight was recorded every week. Rats which had blood sugar values ≥11.6 mmol/L were used for this study. 1 week diabetic rats were treated with curcumin (Cur; 100 or 200 mg/kg/day P.O [9]) or vehicle for 16 weeks. All the animals were provided with food and water *ad libitum*.

### Analysis of Left Ventricular Function

Animals were placed under light anesthesia with pentobarbital sodium by the same doctor using echocardiographic system (GE logiq5 pro) equipped with a 12-MHz linear probe. Derived echocardiography parameters included heart rate, left ventricular end-diastolic diameter (LVEDD), left ventricular end-systolic diameter (LVESD) and interventricular septal diastolic wall thickness (IVSD). To assess left ventricular systolic function, fractional shortening (FS) and ejection fraction (EF) were calculated as follows: FS =  [(LVEDD-LVESD)/LVEDD] ×100% [10], EF = [(LVEDD^3^-LVEDS^3^)/LVEDD^3^] ×100% [11].

### Measurement of Myocardial Cell Size

The hearts were arrested with 15% KCl, fixed with 4% paraformaldehyde, sectioned into 2 mm thick transverse slices and parallel to the atrioventricular ring, then embedded in paraffin, and cut into 4 µm slices [12]. The sections were stained with hematoxylin-eosin (H&E). Short-axis diameter of cardiac myocyte was measured for 10 myocytes selected per section at 400-fold magnification by light microscopy. Each average value was obtained based on the data from 10 myocytes and was used as an independent sampling data [13].

### Measurement of Myocardial fibrosis

Paraffin-embedded tissue sections were stained with masson’s trichrome. The area of myocardial fibrosis was quantified by a color image analyzer (HPIAS-1000, Wuhan, China), using the difference in color (blue fibrotic area as opposed to red myocardium). The extent of myocardial fibrosis was taken as the ratio of the fibrosis area to the whole area of the myocardium [13].

### Electron Microscopy Detection

About 1 mm^3^ of tissues was obtained from left ventricular, immediately prefixed in 2.5% glutaraldehyde for 2 h, and then rinsed in phosphate-buffered saline (0.1 mol/L, pH 7.4). The tissues were then fixed in 1% osmium tetroxide and rinsed in phosphate-buffered saline (0.1 mol/L, pH 7.4). Subsequently, the tissues were dehydrated, first in graded ethanol and then in acetone, and finally embedded in Epon 812. Thin sections were cut on an ultramicrotome and double-stained with uranyl acetate and lead citrate. The ultrastructural changes in the heart tissues were observed by transmission electron microscope (FEI Tecnai G^2^12).

### Measurement of Myocardial Enzymes and Inflammatory Cytokines in Serum

After echocardiography measurements, the blood samples were collected from the abdominal artery and the serum was separated. Creatine kinase-MB (CK-MB), lactate dehydrogenase (LDH) and aspartate amino transferase (AST) were determined by auto-biochemical analysis system (OLYPUS AU2700). Interleukin-1 beta (IL-1β) and tumor necrosis factor-alpha (TNF-α) in serum were determined by enzyme-linked immunosorbent assay (ELISA) kits (R&D ELISA Kit, USA) following the manufacturers instructions.

### Estimation of Superoxide Dismutase (SOD) and Malondialdehyde (MDA) in Heart Tissue

The samples of heart tissue were weighed and homogenized (1∶10, w/v) in 50 mmol·L^−1^ phosphate buffer (pH 7.4). The SOD activity and MDA level were measured by colorimetric analysis using a spectrophotometer with the associated detection kits (Nanking Jiancheng Bioengineering Research Institute, China).

### Terminal Deoxynucleotidyl Transferase-mediated dUTP nick End-labelling (TUNEL) Assay

The paraffin samples were removed from the sections with xylene, rehydrated in graded alcohol series, and then placed in 3% hydrogen peroxide in methanol for 10 min at room temperature. Sections were then incubated with 20 mg·mL^−1^ proteinase K for 15 min. The sections were washed several times in PBS and then incubated with equilibration buffer for 15 s and TdT-enzyme at 37°C for 1 h. Antibody blocking then proceeded for 5 min, and then POD (conjugated with horseradish peroxidase) was dropped on the slides. The 3, 3′-diaminobenzidine (DAB) was used as the staining agent. All these process were performed according to the manufacturer’s instructions (Roche Applied Science, USA).

### Immunohistochemical Determination of Advanced Glycation End Products (AGEs), Bcl-2, Bax and Caspase-3

Tissue sections were incubated with primary antibodies (Santa Cruz Biotechnology, USA) of AGEs, Bcl-2, Bax and caspase-3 at 4°C overnight, and then incubated with biotinylated horse anti-mouse IgG for 30 min and thereafter with the avidin-biotin-peroxidase complex. The reaction was visualized with DAB solution. After counterstaining with hematoxylin, the slides were dehydrated and mounted in Permount. Images (×400 magnification) were captured. The staining for AGEs was quantified with Image-Pro Plus v6 analysis software.

### Assay of Caspase-3 Activity

Caspase-3 activity in heart was quantified according to Caspase-3 activity assay kit (Beyotime Institute of Biotechnology, China), which detects the production of the chromophore p-nitroanilide after its cleavage from the peptide substrate DEVD-p-nitroanilide.

### Measurements of Rac1 Activity

Rac1 activation was performed according to the manufacturer’s protocol (Rac1 Activation Assay Kit; Millipore, USA). 1×MLB (Mg^2+^lysis/wash buffer) with Protease Inhibitor Cocktail (sigma) was used for protein extract. Sample homogenates were spun at 12,000 rpm for 15 min and the supernatants were saved for active Rac1 pull-down assay and for total Rac1 content. Protein concentrations were measured with the BCA protein assay kit to allow equal loading of protein. For each sample, 15 µL of PAK1-PBD agarose beads was added and incubated 1 h at 4°C with gentle agitation. The agarose beads were collected by spinning at 14,000 g for 1 min at 4°C and the supernatants removed. Precipitated complexes were washed three times with 1×MLB. Following washing, the agarose beads were resuspended in 40 µL of 2×Laemmli buffer and boiled for 5 min. Proteins were separated by 10% SDS-PAGE, transferred onto nitrocellulose membrane, and detected by immunoblotting using an anti-Rac1-specific antibody.

### Measurement of NADP^+^/NADPH Ratio

The NADP^+^ and NADPH were determined using EnzyChrom™ NADP^+^/NADPH assay kit (Bioassay Systems, CA). Manufacturer’s protocols were followed. It based on a glucose dehydrogenase cycling reaction. The intensity of the reduced product color, measured at 565 nm, is proportionate to the ratio of NADP^+^/NADPH in the sample.

### RT-PCR Analysis of Receptor for Advanced Glycation End Products (RAGE) mRNA Expression

Total RNA was extracted from heart tissue using TRIzol reagent according to the manufacturer’s protocol (Promega, Madison, WI, USA) and the RNA concentrations were determined by UV spectrophotometry. Reverse transcription for cDNA synthesis was performed with 2 µg total RNA using RevertAid™ First Strand cDNA Synthesis Kits (Fermentas, Hanover, MD, USA). The RAGE primers (288bp): 5′-ATAGCCGCTCTGCTCA TTGG-3′ (forward) and 5′-ATCATGTGGGCTCTGGTTGG-3′ (reverse); The GAPDH primers (451bp): 5′-TGTGAACGGATTTGGCCGTA-3′ (forward) and 5′-TAAGCAGTTGGTGGTGC. AGG-3′ (reverse). The semi-quantitative polymerase chain reaction (PCR) reaction was performed on an Applied Biosystems model Fast PCR System with Universal PCR Master Mix (Applied Biosystems, Framingham, MA, USA) in a total volume of 50 µL. The condition for PCR was as follows: 95°C for 3 min, followed by 35 cycles of 95°C for 30 s, 60°C for 30 s and 72°C for 30 s. GAPDH, which was uniformly expressed among all samples, was used as an endogenous reference gene. Fold change in intensity of the target gene was calculated using Gene-tool version 2.0.

### Western Blot Analysis

Frozen left ventricular tissue was homogenized in ice-cold lysis buffer [20 mM Tris, pH 7.5, 150 mM NaCl, 1 mM EDTA, 1 mM EGTA, 1% Triton X-100, 2.5 mM sodium pyrophosphate, 1 mM β-glycerolphosphate, 1 mM Na_3_VO_4_, 1 µg/mL aprotinin leupeptin and pepstatin, 1 mM Phenylmethylsulfonyl fluoride (PMSF)] and then centrifuged at 12 000 rpm for 15 min at 4°C. Bicinchoninic Acid (BCA) protein assay (Beyotime, China) was utilized to measure protein concentration in the supernatant. Equal protein was used for Western blot using the following antibodies: RAGE (Bioss, Beijing, China), Bcl-2, Bax, phospho-Akt (Ser473), Akt (Cell Signaling Technology, USA), and gp91^phox^, p47^phox^, phospho-GSK-3β (Ser9), GSK-3β, β-actin (Santa Cruz Biotechnology, USA). The membrane was incubated with horseradish peroxidase-conjugated secondary antibody for 1 h at 37°C. Blots were developed by ECL kit (Pierce Biosciences, USA).

### Statistical Analysis

Data were presented as mean ± SEM. Statistical analysis was performed using a one-way analysis of variance (ANOVA). Differences were considered to be significant at P<0.05.

## Results

### Curcumin Prevented Metabolism Abnormalities

The metabolic characteristics of the experimental animals analyzed in this study are shown in Table 1. In DM group, untreated diabetic rats had markedly lower body weight and higher blood glucose levels compared with the control group (P<0.05). Moreover, heart-to-body weight ratio (HW/BW) in DM group exhibited higher than control group (P<0.05). Compared with the DM group, 200 mg/kg curcumin-treated group gained significantly higher body weight and lower HW/BW (P<0.05). Furthermore, all doses of curcumin could significantly decrease blood glucose levels in diabetic rats (P<0.05). Whereas, 200 mg/kg curcumin-treated group preserved blood glucose levels within normal range. In the basal fasting state, DM group showed significantly higher TG and TC than control group (P<0.05), and TG was markedly decreased in diabetic rats treated with 200 mg/kg curcumin (P<0.05 vs DM group). However, no significant differences in TC were observed between DM group and curcumin-treated group.

**Table 1:** Curcumin (Cur) prevented metabolism abnormalities.

Group	Body weight(g)	HW/BW(mg/g)	Blood glucose(mmol/L)	TG(mmol/L)	TC(mmol/L)
**Control**	401±18	2.67±0.14	5.1±0.1	0.80±0.08	1.24±0.06
**DM**	247±19*	4.05±0.28*	20.6±1.6*	1.19±0.14*	1.49±0.07*
**DM+Cur100** **mg/kg**	293±25	3.52±0.16	13.1±2.2#	1.08±0.08	1.35±0.11
**DM+Cur200** **mg/kg**	332±27#	3.21±0.12#	8.7±1.7#	0.85±0.11#	1.39±0.08

TG: triglycerides; TC: total cholesterol; Body weight and heart weight were measured on the day the rat was killed. Blood glucose, TG and TC levels were measured in the basal fasting state on the day the rat was killed. Data are means ± SEM;

* *P*<0.05 vs control group;

# *P*<0.05 vs DM group; n = 8–10 per group.

### Curcumin Alleviated DM-induced Left Ventricular Disfunction

Cardiac performance parameters derived from echocardiography are shown in Table 2. DM group had a slower heart rate than control group, while 200 mg/kg curcumin-treated group caused a marked increase in heart rate compared to DM group (P<0.05). IVSD exhibited higher in DM group than control group (P<0.05), and 100 or 200 mg/kg curcumin-treated group showed lower IVSD than that of DM group (P<0.05), which indicated that curcumin could inhibit interventricular septal hypertrophy. FS and EF, the index of left ventricular systolic function, were significantly decreased in DM group. And compared with DM group, these changes were attenuated when diabetic rats were treated with 200 mg/kg curcumin(P<0.05).

**Table 2:** Curcumin (Cur) alleviated DM-induced left ventricular disfunction.

Group	Heart rate (bpm)	IVSD(mm)	LVEDD(mm)	LVESD(mm)	FS(%)	EF(%)
**Control**	378±11	1.32±0.07	2.78±0.12	1.53±0.12	45.1±2.5	82.4±2.3
**DM**	308±15^*^	1.88±0.11^*^	3.35±0.30	2.25±0.24^*^	33.1±3.8^*^	68.5±5.2^*^
**DM+Cur100** **mg/kg**	330±13	1.36±0.08#	2.72±0.16	1.82±0.12	36.7±3.2	76.6±2.9
**DM+Cur200** **mg/kg**	364±17#	1.35±0.08#	2.85±0.20	1.60±0.15#	44.0±2.8#	81.8±2.7#

IVSD, interventricular septal diastolic wall thickness; LVEDD, left ventricular end diastolic diameter; LVESD, left ventricular end systolic diameter; FS, fractional shortening = [(LVEDD-LVESD)/LVEDD] ×100%; EF, ejection fraction = [(LVEDD^3^-LVESD^3^)/LVEDD^3^] ×100%. Data are means ± SEM;

*P*<0.05 vs control group;

# *P*<0.05 vs DM group; n = 6 per group.

### Curcumin Attenuated DM-induced Cardiomyocyte Hypertrophy and Interstitial fibrosis

**Figure 1:**
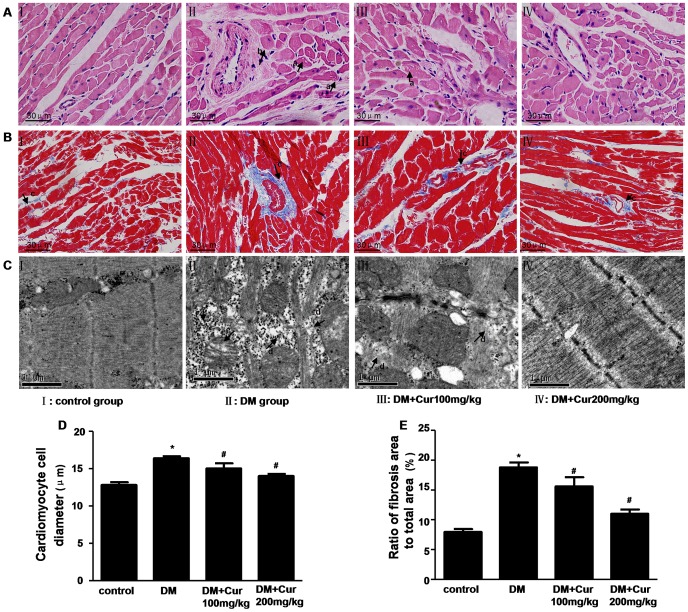
Curcumin attenuated pathological change in the heart of experimental diabetic rats. A: Representative pictures of myocardial tissue sections stained with hematoxylin and eosin (magnification = 400×). n = 6 per group. Arrow ***a*** indicates perinuclear vacuolization; arrow ***b*** indicates cardiomyocyte hypertrophy; B: Representative pictures of myocardial tissue sections stained with masson’s trichrome (magnification = 400×). n = 6 per group. Arrow ***c*** indicates myocardial fibrosis stained in blue; C: Representative transmission electron micrographs of left ventricular specimens. n = 3 per group. Arrow ***d*** indicates destruction and loss of myofibrils; arrow ***e*** indicates swollen mitochondria; arrow ***f*** indicates a number of glycogen lysis; D: Quantitative analysis of cardiomyocyte cell diameter. n = 6 per group; E: Quantitative analysis of fibrosis area. n = 6 per group. Data are mean ± SEM.^ *^ *P*<0.05 vs control group; ^#^ *P*<0.05 vs DM group.

The myocardial structural was examined by H&E staining, masson’s trichrome staining and transmission electron microscope, respectively. As shown in Fig. 1A and C, well organized, typical symmetric myofibrils comprised of Z lines with sarcomeres, packed mitochondria beside the fibers were seen in control rats. In contrast, perinuclear vacuolization, destruction and loss of myofibrils over sarcomere units, swollen mitochondria and a number of glycogen lysis were evident in DM group rats. Curcumin treatment normalized alterations in myofilaments, Z-lines and mitochondria, along with reduced lysis of glycogen in diabetic rats.

Particularly, cell size was measured to evaluate the cardiomyocyte hypertrophy of left ventricle. As shown in Fig. 1D, cardiomyocyte diameter was significantly greater in DM group versus control group (P<0.05). In contrast to DM group, 100 or 200 mg/kg curcumin-treated group improved the diabetes-induced cardiomyocyte hypertrophy (P<0.05).

Additionally, obvious fibrosis in the heart was shown in DM group rats, with a no uniform pattern, as well as destroyed and disorganized collagen network structure in the interstitial and perivascular areas. However, the fibrotic changes in the heart were significantly mitigated when diabetic rats treated with 100 or 200 mg/kg curcumin (Fig. 1B and E, P<0.05).

### Curumin Inhibited AGEs Accumulation and RAGE Expression

**Figure 2:**
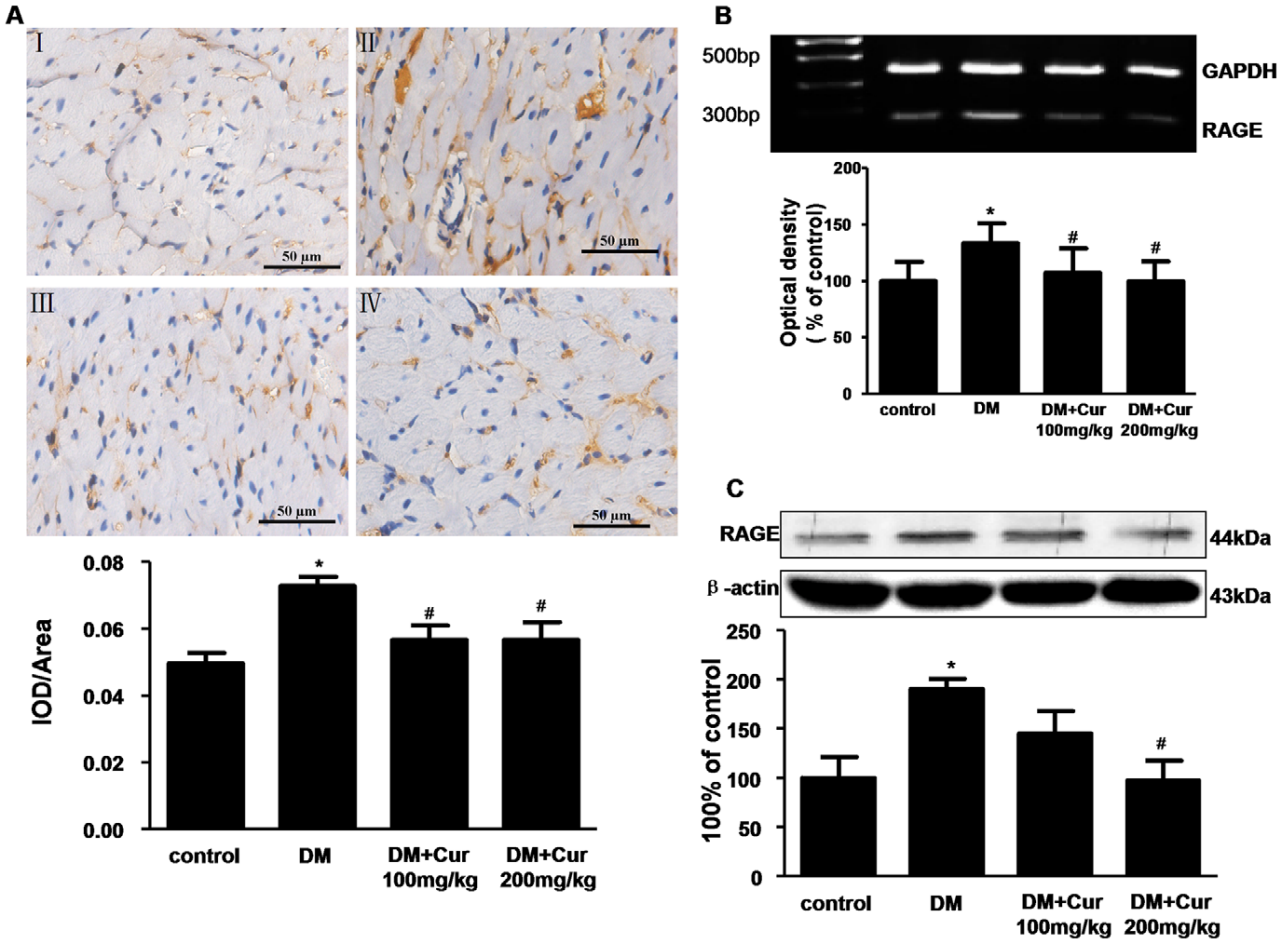
Curumin inhibited AGEs accumulation and RAGE expression in the heart of experimental diabetic rats. A: Representative immunohistochemical staining and quantitative analysis of AGEs. n = 5 per group. AGEs: advanced glycation end products. B: PCR analysis of RAGE mRNA expression. n = 3 per group. C: western blots analysis of RAGE. RAGE: receptor for advanced glycation end products. n = 3 per group. Data are mean ± SEM. ^*^ *P*<0.05 vs control group; ^#^ *P*<0.05 vs DM group.

AGEs are the non-enzymatic results of a chain of non-oxidative and oxidative reactions of excessive sugars with proteins and/or lipids [14]. RAGE mediates the effects of AGEs. AGEs-RAGE interaction contributes to diabetic cardiomyopathy by cross-linking myocardial proteins such as collagen and elastin, and by promoting collagen accumulation [15]. In the present study, AGEs formation was more prominent in DM group rats (Fig. 2A, P<0.05). In addition, there was marked increase in RAGE mRNA expression (Fig. 2B) and RAGE protein level (Fig. 2C) in DM group compared to control group (P<0.05). Curcumin treatment hampered the diabetes-induced AGEs accumulation and RAGE expression (P<0.05 vs DM group).

### Curumin Inhibited Myocardial Injury, Inflammation and Oxidative Stress

**Figure 3:**
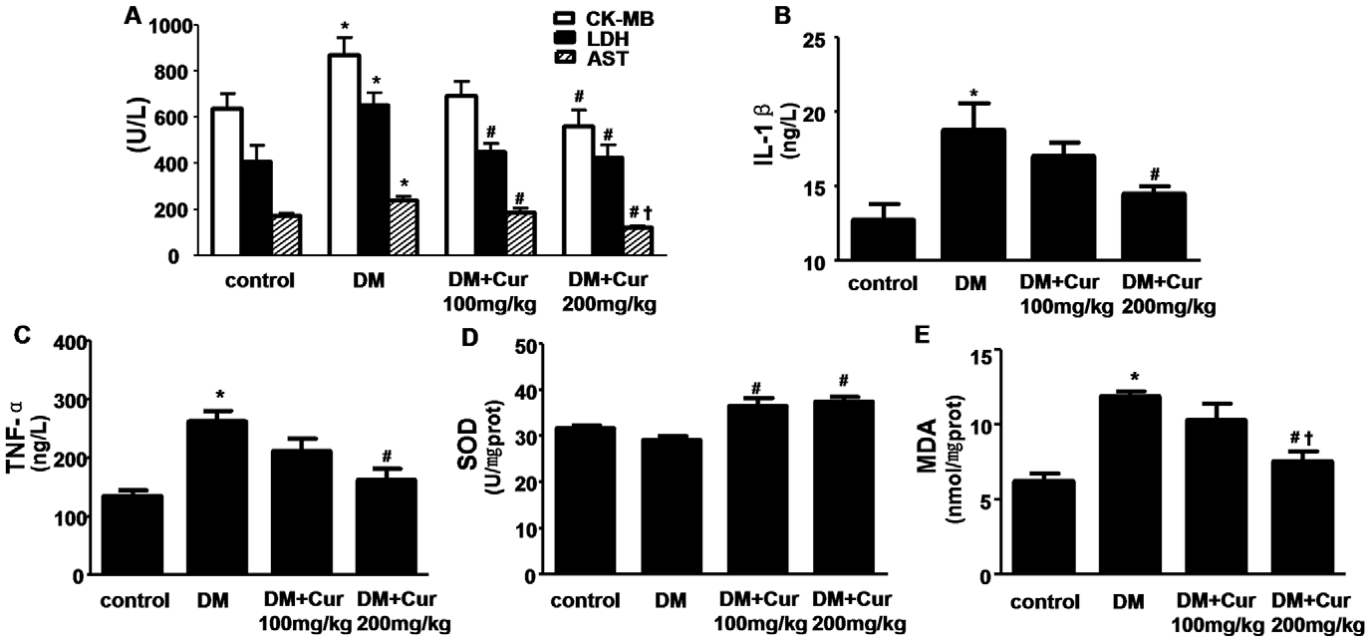
Curumin inhibited myocardial injury, inflammation and oxidative stress in experimental diabetic rats. A: Curumin inhibited serum myocardial enzyme release. CK-MB: creatine kinase-MB; LDH: Lactate dehydrogenase; AST: aspartate amino transferase; B: Curumin reduced the levels of IL-1β in serum. IL-1β: interleukin-1beta. C: Curumin reduced the levels of TNF-α in serum. TNF-α: tumor necrosis factor alpha. D: Curumin increased SOD activity in heart tissue. SOD: superoxide dismutase. E: Curumin decreased MDA content in heart tissue. MDA: malondialdehyde. Data are mean ± SEM.^ *^ *P*<0.05 vs control group; ^#^ *P*<0.05 vs DM group; **^†^** *P*<0.05 vs DM+Cur100 mg/kg group. n = 8–10 per group.

Myocardial enzymes CK-MB, LDH and AST are biochemical indicators of myocardial injury (Fig. 3A). As compared with control group, the levels of all three enzymes were significantly elevated in the DM group (P<0.05). Curcumin protected diabetic rats against cardiac injury, which was evidenced by decreased myocardial enzymes in 200 mg/kg curcumin-treated group (P<0.05 vs DM group).

Moreover, the inflammatory factors IL-1β and TNF-α were also increased in DM group compared to control group (P<0.05). By contrast, lower levels of IL-1β and TNF-α were found in 200 mg/kg curcumin-treated group compared to DM group (Fig. 3B and C, P<0.05).

There was increased accumulation of lipid peroxides with concordant increase content of MDA (Fig. 3D) and decrease activity of SOD (Fig. 3E) in hearts of DM group rats(P<0.05 vs control group). Diabetic rats treatment with 200 mg/kg curcumin markedly inhibited MDA content and up-regulated the activity of SOD (P<0.05 vs DM group).

### Curcumin Attenuated NADP^+^/NADPH Ratio, Rac1 Activity and the Expression of NADPH Oxidase Subunits of gp91 *^phox^*, p47 *^phox^* in Diabetic Rats

**Figure 4:**
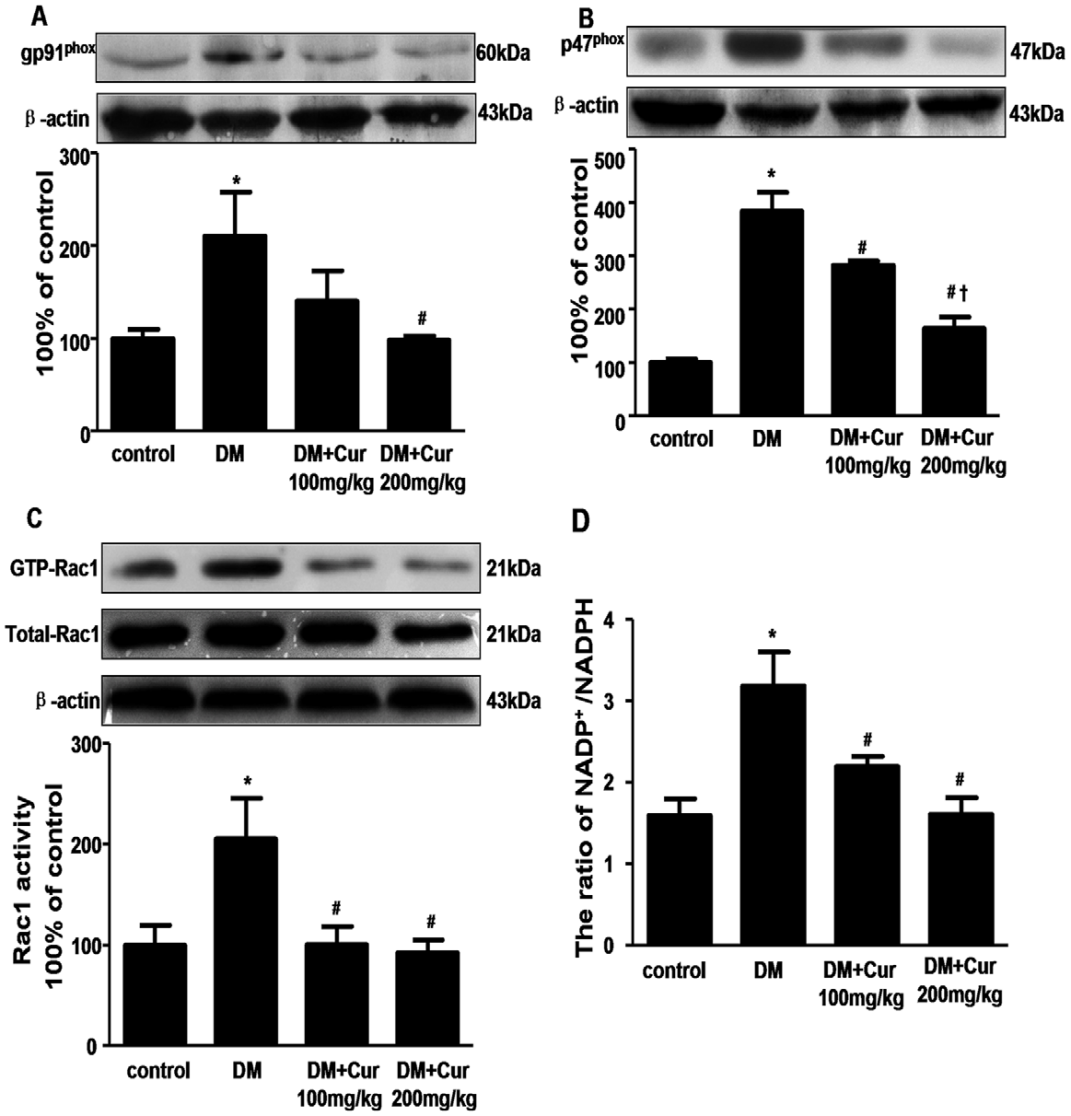
Curcumin decreased the expression of NADPH oxidase subunits of gp91^phox^ and p47^phox^, Rac1 activity and the ratio of NADP^+^/NADPH in the heart of experimental diabetic rats. A: western blots analysis of gp91^phox^. n = 3 per group; B: western blots analysis of p47^phox^. n = 3 per group; C: Rac1 activity assay. n = 3 per group; D: the ratio of NADP^+^/NADPH. n = 6 per group. Data are mean ± SEM.^ *^ *P*<0.05 vs control group; ^#^ *P*<0.05 vs DM group; ^†^ *P*<0.05 vs DM+Cur100 mg/kg group.

NADPH oxidase is an important enzymatic system responsible for myocardial ROS production [16]. It is a membrane-associated enzyme that transfers reducing equivalents from NADPH or NADH to oxygen, which results in superoxide anion (O^2−^) generation. This enzyme complex comprises two membrane subunits (gp91 *^phox^* and p22 *^phox^*) and four cytosolic subunits (p40*^phox^*, p47*^phox^*, p67*^phox^*, and Rac1) [17]. We assessed changes in NADP^+^/NADPH ratio, Rac1 activity and the protein expression of gp91*^phox^*, p47*^phox^* in the hearts. As shown in Fig. 4, the ratio of NADP^+^/NADPH and Rac1 activity were significantly higher in DM group than those in control group. Compared with DM group, the ratio of NADP^+^/NADPH and Rac1 activity in 100 or 200 mg/kg curcumin-treated group were significantly lower than those of DM group (P<0.05). Moreover, increased expression of NADPH subunits gp91*^phox^* and p47*^phox^* were found in DM group compared to control group, which were significantly reduced in 200 mg/kg curcumin-treated groups (P<0.05).

### Curcumin Prevented DM-induced Cardiomyocytes Apoptosis

**Figure 5:**
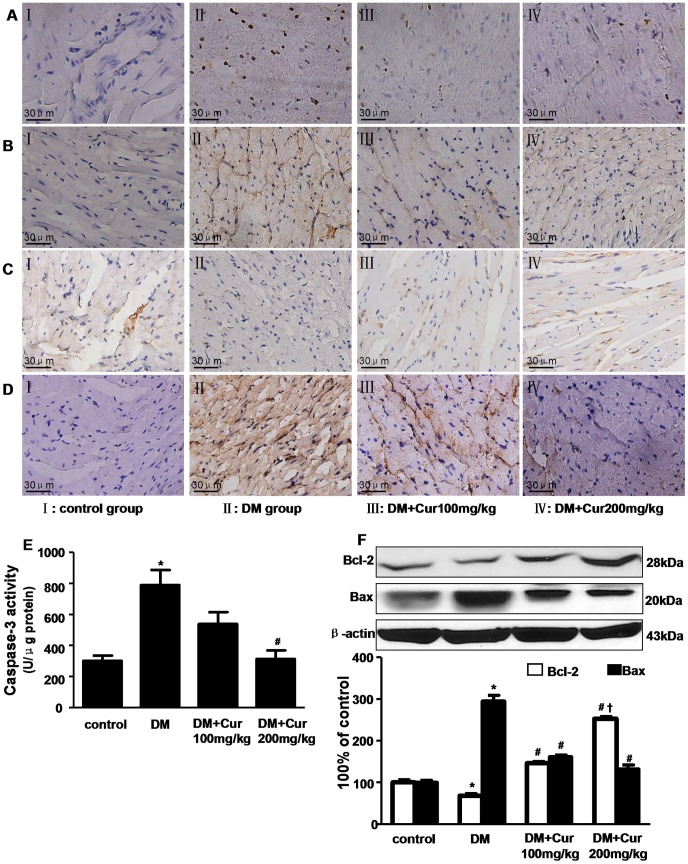
Curcumin reduced apoptosis in the heart of experimental diabetic rats. A: Representative pictures of the TUNEL assay. n = 3 per group; B, C and D: Representative immunohistochemical staining of Caspase-3, Bcl-2 and Bax expression, respectively. n = 5 per group. magnification = 400×; E: Caspase-3 activity assay. n = 6 per group; F: western blots analysis of Bcl-2 and Bax expression. n = 3 per group. Data are mean ± SEM. ^*^ *P*<0.05 vs control group; ^#^ *P*<0.05 vs DM group; **^†^** *P*<0.05 vs DM+Cur100 mg/kg group.

As shown in Fig. 5A, apoptotic cells were stained in brown by TUNEL staining. The apoptotic cells in DM group were much higher than control group. And compared with DM group, curcumin treatment significantly reversed DM-induced myocardial cell apoptosis in diabetic rats.

The expression of anti-apoptotic protein Bcl-2, pro-apoptotic protein Bax and caspase-3 was assessed by immunohistochemistry. As shown in Fig. 5B–D, enhanced expression of Bax and caspase-3 but reduced expression of Bcl-2 was presented in DM group in contrast to control group. Remarkably, diabetic rats treated with 200 mg/kg curcumin could significantly up-regulate Bcl-2 expression and down-regulate Bax, caspase-3 expression. This was further confirmed by assay of caspase-3 activity (Fig. 5E) and western bolt analysis of Bcl-2 and Bax expression (Fig. 5F).

### Curcumin Activated Akt/GSK-3β Signaling Pathway

**Figure 6:**
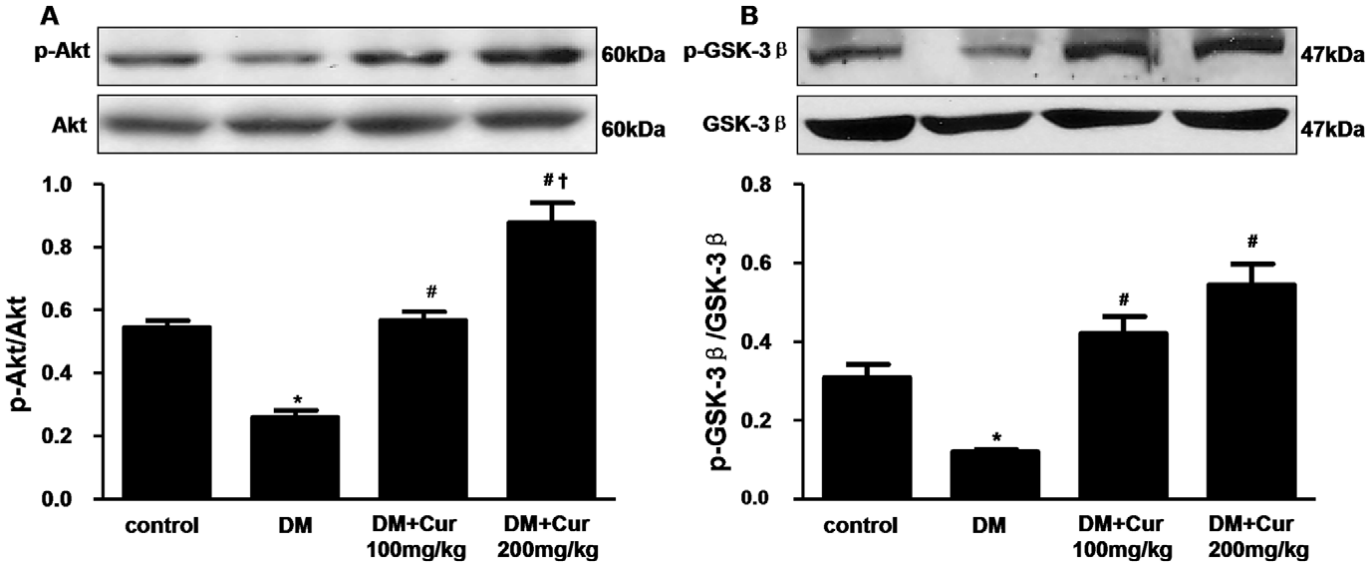
Curcumin activated Akt and inactivated GSK-3β in the heart of experimental diabetic rats. A: western blots analysis of p-Akt and Akt. B: western blots analysis of p-GSK-3β and GSK-3β. Data are mean ± SEM. n = 3 per group. ^*^ *P*<0.05 vs control group; ^#^ *P*<0.05 vs DM group; **^†^** *P*<0.05 vs DM+Cur100 mg/kg group.

It is well known that Akt plays a critical role in regulating and controlling glycogen synthesis, cell growth and survival. GSK-3β is a critical downstream element of Akt whose activity can be inhibited by Akt-mediated phosphorylation at Ser9. To further elucidate the potential mechanism of cardioprotective effects for curcumin, we investigated the effects of curcumin on the Akt/GSK-3β signaling pathway. Our results revealed that Akt phosphorylation was significantly hampered in the diabetic hearts (Fig. 6A) by western blot analysis. In addition, there was marked activation of GSK-3β in the diabetic hearts (Fig. 6B). In contrast,100 or 200 mg/kg curcumin induced a significant increase in phosphorylation of Akt and GSK-3β in myocardium (P<0.05). There was no significant difference for total Akt and GSK-3β levels in all groups.

## Discussion

Curcumin is the most abundant polyphenol present in the dietary spice turmeric. In addition to extensive use as food additive, curcumin has been shown to exert anti-inflammatory and antioxidant effects in various acute and chronic diseases such as atherosclerosis, cancer, hepatic diseases and so on. Several studies have demonstrated that curcumin is atoxic, also in very high doses. Treatment of humans during three months with 8,000 mg curcumin per day showed no side effects [18]. In the most recent years the increasing interest for curcumin in the prevention/treatment of diabetes and associated complications has arose.

There are several forms of diabetes, including type-1 diabetes (insulin-dependent), type-2 diabetes (noninsulin-dependent), and gestational diabetes. Experimental diabetes produced by low dose of STZ combined with high energy intake is regarded as a general strategy to obtain type 2 diabetes animal model, since it simulates the real course of human type 2 diabetes mellitus [19], [20]. The high energy diet induces insulin resistance at first. And then an injection of low dose STZ makes partial dysfunction of beta cell to suppress insulin secretion, which works as a compensation to insulin resistance. In the present study, a low dose of STZ injection (40 mg/kg body weight) after 8-week feeding of the high-energy diet has exerted a great effect to induce diabetes by markedly elevating serum glucose, TG and TC levels. The serum symptoms induced by this way were more similar to those of type 2 diabetes than those of type 1 diabetes.

Diabetic cardiomyopathy defined as ventricular dysfunction with increased risk of heart failure, in the absence of hypertension, coronary artery and valvular heart diseases [21], is frequently seen in both humans and animals. Consistently with pervious reports, the 16 weeks untreated diabetic rats in the present study were characterized by declined diastolic and systolic myocardial performance, which associated with excess collagen accumulation and cardiac hypertrophy. In addition, there are increased AGEs accumulation coupled with elevated expression of RAGE, enhanced myocardial expression of NADPH oxidase isoforms gp91^phox^, p47^phox^ and Rac1 activity, attenuated antioxidant defense (decreased SOD activity) coupled with increased myocardial lipid peroxidation, inactivation of pro-survival pathways of Akt/GSK-3β, and eventually culminating in cell death, cardiac fibrosis and increased inflammation in the untreated diabetic heart. In contrast, we have demonstrated that long-term administration of curcumin prevents the development of such characteristic alterations of DCM. And the beneficial effects of curcumin could be explained in part as follows: firstly, curcumin treatment abrogated the elevated blood glucose and triglyceride. Previous studies have demonstrated that the onset of cardiovascular complications could be retarded by control of metabolic abnormalities [22]. Our results further confirmed that strict glycemic control may play a central role in progression of DCM. Secondly, curcumin attenuated the oxidative stress. Curcumin has the ability to intercept and neutralize ROS (superoxide, peroxyl, hydroxyl radicals) and NOS (NO, peroxynitrite) [23]. Its antioxidant capacity is 100-fold stronger than that of vitamin E/C [24]. Oxidative stress is defined as the imbalance between the production and the elimination of free radicals, which plays a critical role in the development of heart failure and left ventricular remodeling in DCM [25]. Hyperglycemia exacerbates glucose oxidation and mitochondrial generation of reactive oxygen species (ROS) [26], which causes DNA damage and contributes to accelerated apoptosis. NADPH oxidase is a critical determinant of myocardial ROS generation [16]. In this report, we have demonstrated that curcumin decreased expression of NADPH oxidase subunits gp91*^phox^* and gp47*^phox^*, down-regulated Rac1 activity, restored SOD activity and reduced lipid peroxidation in diabetic hearts. Thirdly, curcumin suppressed AGEs accumulation and the expression of RAGE. A dramatic increase in the abundance of RAGE was observed in diabetic patients with high levels of blood AGEs [27], [28]. Hyperglycaemia facilitates the formation of AGEs. The interaction of AGEs and RAGE may stimulate the activation of a diverse array of signaling cascades associated with increased oxidative stress, cell growth and inflammation, including MAPKs, PI3K and NF-κB signalling pathway [29]. AGEs-RAGE interaction mediates the development of diabetic complications, which was confirmed in studies with RAGE^−/−^ mice [14]. Similar effect of curcumin on AGEs-RAGE was supported by previous studies that curcumin inhibited gene expression of RAGE in hepatic stellate cells by elevating PPARg activity and attenuating oxidative stress [30]. Fourthly, curcumin is also a potent inhibitor of tissue growth factor beta (TGF-β) and fibrogenesis [31]. Previous studies have suggested that curcumin have positive effects in diseases such as kidney fibrosis, lung fibrosis, liver cirrhosis and in prevention of formation of tissue adhesions [32]. As we known, one of the crucial hallmarks of DCM is fibrosis [33]. Our results indicated that curcumin markedly prevented cardiac fibrosis in experimental diabetes. Fifthly, cardiac inflammation, characterized by increased levels of pro-inflammatory cytokines, was suppressed by curcumin as well. Pro-inflammatory cytokines, such as IL-1β and TNF-α, critically participate in the manifestation of DCM [34]. Curcumin has been used as an antiinflammation agent for generations. Accumulating evidences have approved that curcumin is a strong NF-kB, COX-2, LOX and iNOS inhibitor and against stress-induced overinflammation [35], [36]. Finally, curcumin activated the important pro-survival signaling pathway Akt/GSK3β in diabetic hearts. Akt promotes cell survival by inhibiting several targets involved in apoptotic signaling cascades. GSK-3β, a major substrate of Akt, not only have central functions in glycogen metabolism and insulin action, but also plays a crucial role in transmitting the apoptotic signal [37], [38]. In diabetes, Akt phosphorylation could be reduced by elevated circulation of free fatty acids and inflammatory cytokines, which leads to defective glucose transport and insulin resistance. Additionally, Akt has a clearly defined role in regulating cardiovascular functions such as cardiac growth, contractile function and coronary angiogenesis [39]. Our results demonstrated that Akt/GSK-3β pathways involved in mediating the effects of curcumin on DCM. In all, it is noteworthy to mention that every point referred above does not work independently but interrelate tightly.

Taken together, our study strongly suggests that curcumin may have great therapeutic potential in the treatment of DCM, and perhaps other cardiovascular disorders by ameliorating metabolic abnormalities, oxidative stress, AGEs-RAGE interaction, fibrosis, inflammation and cell death pathways.

## References

[1] Shaw JE, Sicree RA, Zimmet PZ (2010) Global estimates of the prevalence of diabetes for 2010 and 2030. Diabetes Res Clin Pract 87: 4–14. 19896746 10.1016/j.diabres.2009.10.007

[2] Westermann D, Walther T, Savvatis K, Escher F, Sobirey M, et al (2009) Gene deletion of the kinin receptor B1 attenuates cardiac inflammation and fibrosis during the development of experimental diabetic cardiomyopathy. Diabetes 58: 1373–1381. 19276445 10.2337/db08-0329 PMC2682670

[3] Huynh K, McMullen JR, Julius TL, Tan JW, Love JE, et al (2010) Cardiac-specific IGF-1 receptor transgenic expression protects against cardiac fibrosis and diastolic dysfunction in a mouse model of diabetic cardiomyopathy. Diabetes 59: 1512–1520. 20215428 10.2337/db09-1456 PMC2874713

[4] Falcao-Pires I, Leite-Moreira AF (2012) Diabetic cardiomyopathy: understanding the molecular and cellular basis to progress in diagnosis and treatment. Heart Fail Rev 17: 325–44. 21626163 10.1007/s10741-011-9257-z

[5] Shehzad A, Ha T, Subhan F, Lee YS (2011) New mechanisms and the anti-inflammatory role of curcumin in obesity and obesity-related metabolic diseases. Eur J Nutr 50: 151–161. 21442412 10.1007/s00394-011-0188-1

[6] Sharma S, Chopra K, Kulkarni SK (2007) Effect of insulin and its combination with resveratrol or curcumin in attenuation of diabetic neuropathic pain: participation of nitric oxide and TNF-alpha. Phytother Res 21: 278–283. 17199240 10.1002/ptr.2070

[7] Wang HJ, Jin YX, Shen W, Neng J, Wu T, et al (2007) Low dose streptozotocin (STZ) combined with high energy intake can effectively induce type 2 diabetes through altering the related gene expression. Asia Pac J Clin Nutr 16 Suppl 1 412–417. 17392141

[8] Sharma AK, Bharti S, Ojha S, Bhatia J, Kumar N, et al (2011) Up-regulation of PPARgamma, heat shock protein-27 and -72 by naringin attenuates insulin resistance, beta-cell dysfunction, hepatic steatosis and kidney damage in a rat model of type 2 diabetes. Br J Nutr 106: 1713–1723. 21736771 10.1017/S000711451100225X

[9] El-Moselhy MA, Taye A, Sharkawi SS, El-Sisi SF, Ahmed AF (2011) The antihyperglycemic effect of curcumin in high fat diet fed rats. Role of TNF-alpha and free fatty acids. Food Chem Toxicol 49: 1129–1140. 21310207 10.1016/j.fct.2011.02.004

[10] Salloum FN, Chau VQ, Hoke NN, Abbate A, Varma A, et al (2009) Phosphodiesterase-5 inhibitor, tadalafil, protects against myocardial ischemia/reperfusion through protein-kinase g-dependent generation of hydrogen sulfide. Circulation 120: S31–S36. 19752383 10.1161/CIRCULATIONAHA.108.843979 PMC4230451

[11] Lin YC, Leu S, Sun CK, Yen CH, Kao YH, et al (2010) Early combined treatment with sildenafil and adipose-derived mesenchymal stem cells preserves heart function in rat dilated cardiomyopathy. J Transl Med 8: 88. 20868517 10.1186/1479-5876-8-88 PMC2956711

[12] Oudit GY, Crackower MA, Eriksson U, Sarao R, Kozieradzki I, et al (2003) Phosphoinositide 3-kinase gamma-deficient mice are protected from isoproterenol-induced heart failure. Circulation 108: 2147–2152. 12963636 10.1161/01.CIR.0000091403.62293.2B

[13] Gurusamy N, Watanabe K, Ma M, Zhang S, Muslin AJ, et al (2005) Inactivation of 14–3–3 protein exacerbates cardiac hypertrophy and fibrosis through enhanced expression of protein kinase C beta 2 in experimental diabetes. Biol Pharm Bull 28: 957–962. 15930726 10.1248/bpb.28.957

[14] Bierhaus A, Humpert PM, Morcos M, Wendt T, Chavakis T, et al (2005) Understanding RAGE, the receptor for advanced glycation end products. J Mol Med (Berl) 83: 876–886. 16133426 10.1007/s00109-005-0688-7

[15] Campbell DJ, Somaratne JB, Jenkins AJ, Prior DL, Yii M, et al (2011) Impact of type 2 diabetes and the metabolic syndrome on myocardial structure and microvasculature of men with coronary artery disease. Cardiovasc Diabetol 10: 80. 21929744 10.1186/1475-2840-10-80 PMC3182888

[16] Frantz S, Brandes RP, Hu K, Rammelt K, Wolf J, et al (2006) Left ventricular remodeling after myocardial infarction in mice with targeted deletion of the NADPH oxidase subunit gp91PHOX. Basic Res Cardiol 101: 127–132. 16273323 10.1007/s00395-005-0568-x

[17] Peng T, Lu X, Feng Q (2005) Pivotal role of gp91phox-containing NADH oxidase in lipopolysaccharide-induced tumor necrosis factor-alpha expression and myocardial depression. Circulation 111: 1637–1644. 15795323 10.1161/01.CIR.0000160366.50210.E9

[18] Chainani-Wu N (2003) Safety and anti-inflammatory activity of curcumin: a component of tumeric (Curcuma longa). J Altern Complement Med 9: 161–168. 12676044 10.1089/107555303321223035

[19] Wang HJ, Jin YX, Shen W, Neng J, Wu T, et al (2007) Low dose streptozotocin (STZ) combined with high energy intake can effectively induce type 2 diabetes through altering the related gene expression. Asia Pac J Clin Nutr 16 Suppl 1 412–417. 17392141

[20] Ti Y, Xie GL, Wang ZH, Bi XL, Ding WY, et al (2011) TRB3 gene silencing alleviates diabetic cardiomyopathy in a type 2 diabetic rat model. Diabetes 60: 2963–2974. 21933987 10.2337/db11-0549 PMC3198078

[21] Tarquini R, Lazzeri C, Pala L, Rotella CM, Gensini GF (2011) The diabetic cardiomyopathy. Acta Diabetol 48: 173–181. 20198391 10.1007/s00592-010-0180-x

[22] Boudina S, Abel ED (2007) Diabetic cardiomyopathy revisited. Circulation 115: 3213–3223. 17592090 10.1161/CIRCULATIONAHA.106.679597

[23] Jovanovic SV, Boone CW, Steenken S, Trinoga M, Kaskey RB (2001) How curcumin works preferentially with water soluble antioxidants. J Am Chem Soc 123: 3064–3068. 11457017 10.1021/ja003823x

[24] Ruby AJ, Kuttan G, Babu KD, Rajasekharan KN, Kuttan R (1995) Anti-tumour and antioxidant activity of natural curcuminoids. Cancer Lett 94: 79–83. 7621448 10.1016/0304-3835(95)03827-j

[25] Rajesh M, Mukhopadhyay P, Batkai S, Patel V, Saito K, et al (2010) Cannabidiol attenuates cardiac dysfunction, oxidative stress, fibrosis, and inflammatory and cell death signaling pathways in diabetic cardiomyopathy. J Am Coll Cardiol 56: 2115–2125. 21144973 10.1016/j.jacc.2010.07.033 PMC3026637

[26] Wang J, Wang H, Hao P, Xue L, Wei S, et al (2011) Inhibition of aldehyde dehydrogenase 2 by oxidative stress is associated with cardiac dysfunction in diabetic rats. Mol Med 17: 172–179. 20957334 10.2119/molmed.2010.00114 PMC3060979

[27] Brett J, Schmidt AM, Yan SD, Zou YS, Weidman E, et al (1993) Survey of the distribution of a newly characterized receptor for advanced glycation end products in tissues. Am J Pathol 143: 1699–1712. 8256857 PMC1887265

[28] Tanji N, Markowitz GS, Fu C, Kislinger T, Taguchi A, et al (2000) Expression of advanced glycation end products and their cellular receptor RAGE in diabetic nephropathy and nondiabetic renal disease. J Am Soc Nephrol 11: 1656–1666. 10966490 10.1681/ASN.V1191656

[29] Xiang M, Wang J, Zhang Y, Ling J, Xu X (2012) Attenuation of aortic injury by ursolic acid through RAGE-Nox-NFkappaB pathway in streptozocin-induced diabetic rats. Arch Pharm Res 35: 877–886. 22644855 10.1007/s12272-012-0513-0

[30] Lin J, Tang Y, Kang Q, Feng Y, Chen A (2012) Curcumin inhibits gene expression of receptor for advanced glycation end-products (RAGE) in hepatic stellate cells in vitro by elevating PPARgamma activity and attenuating oxidative stress. Br J Pharmacol 166: 2212–2227. 22352842 10.1111/j.1476-5381.2012.01910.x PMC3448888

[31] Gaedeke J, Noble NA, Border WA (2004) Curcumin blocks multiple sites of the TGF-beta signaling cascade in renal cells. Kidney Int 66: 112–120. 15200418 10.1111/j.1523-1755.2004.00713.x

[32] Srinivasan P, Libbus B (2004) Mining MEDLINE for implicit links between dietary substances and diseases. Bioinformatics 20 Suppl 1 i290–i296. 15262811 10.1093/bioinformatics/bth914

[33] Zhou H, Li YJ, Wang M, Zhang LH, Guo BY, et al (2011) Involvement of RhoA/ROCK in myocardial fibrosis in a rat model of type 2 diabetes. Acta Pharmacol Sin 32: 999–1008. 21743486 10.1038/aps.2011.54 PMC4002528

[34] Mano Y, Anzai T, Kaneko H, Nagatomo Y, Nagai T, et al (2011) Overexpression of human C-reactive protein exacerbates left ventricular remodeling in diabetic cardiomyopathy. Circ J 75: 1717–1727. 21519150 10.1253/circj.cj-10-1199

[35] Surh YJ, Chun KS, Cha HH, Han SS, Keum YS, et al (2001) Molecular mechanisms underlying chemopreventive activities of anti-inflammatory phytochemicals: down-regulation of COX-2 and iNOS through suppression of NF-kappa B activation. Mutat Res 480–481: 243–268. 10.1016/s0027-5107(01)00183-x 11506818

[36] Chang DM (2001) Curcumin: a heat shock response inducer and potential cytoprotector. Crit Care Med 29: 2231–2232. 11700434 10.1097/00003246-200111000-00034

[37] Liu Y, Tanabe K, Baronnier D, Patel S, Woodgett J, et al (2010) Conditional ablation of Gsk-3beta in islet beta cells results in expanded mass and resistance to fat feeding-induced diabetes in mice. Diabetologia 53: 2600–2610. 20821187 10.1007/s00125-010-1882-x PMC2991091

[38] Wang Y, Feng W, Xue W, Tan Y, Hein DW, et al (2009) Inactivation of GSK-3beta by metallothionein prevents diabetes-related changes in cardiac energy metabolism, inflammation, nitrosative damage, and remodeling. Diabetes 58: 1391–1402. 19324938 10.2337/db08-1697 PMC2682666

[39] Katare RG, Caporali A, Oikawa A, Meloni M, Emanueli C, et al (2010) Vitamin B1 analog benfotiamine prevents diabetes-induced diastolic dysfunction and heart failure through Akt/Pim-1-mediated survival pathway. Circ Heart Fail 3: 294–305. 20107192 10.1161/CIRCHEARTFAILURE.109.903450 PMC2865995

